# Fabrication and Evaluation of Rapidly Dissolving Microneedles Loaded with Organophosphorus Hydrolase for the Treatment of Transdermal Ethyl Paraoxon Poisoning

**DOI:** 10.3390/pharmaceutics18050567

**Published:** 2026-05-01

**Authors:** Fengqian Cui, Xue Liang, Ming Ma, Yanan Zhai, Jing Gao

**Affiliations:** 1 School of Pharmacy, Qingdao University, Qingdao 266071, China; 2 State Key Laboratory of National Security Specially Needed Medicines, Beijing 100039, China; 3 Academy of Military Medical Sciences, Beijing 100850, China

**Keywords:** organophosphorus hydrolase, transdermal delivery, sulfobutylether-β-cyclodextrin, fast-dissolving microneedle, ethyl paraoxon poisoning

## Abstract

**Background**: Given the severe toxicity of organophosphates and the efficient catalytic hydrolysis of organophosphates by organophosphorus hydrolase (OPH) against them, a rapidly dissolving microneedle patch loaded with OPH offers a promising therapeutic strategy to provide timely detoxification conveniently. **Methods**: The microneedles were prepared with sulfobutylether-β-cyclodextrin (SCD) matrix to load OPHDS5, one of the mutants of OPH, and were characterized in vitro and in vivo to determine tip loading capacity, skin insertion efficiency, and protective efficacy against ethyl paraoxon poisoning. **Results**: Microneedles prepared with 60% *w*/*v* SCD displayed well-defined square-pyramidal tips and a mechanical strength of 4 N/needle at 1200 μm. When containing 1.2 mg OPHDS5, the microneedles enabled SD rats to completely survive a lethal challenge of ethyl paraoxon, achieving a 100% survival rate, significantly alleviating the symptoms resulting from transdermal exposure and markedly attenuating the symptoms resulting from transdermal exposure. **Conclusions**: A rapidly dissolving microneedle patch encapsulating OPH was successfully developed. The patch effectively penetrated the skin and exhibited robust prophylactic and therapeutic activity against ethyl paraoxon poisoning.

## 1. Introduction

Organophosphates (OPs) were first synthesized in the 1930s as agricultural insecticides, and their application was later extended to chemical warfare agents during global conflicts [1]. OPs remain extensively used in developing countries, causing hundreds of thousands of deaths annually from acute poisoning and posing severe threats to agricultural safety and public health [2]. Due to their lipophilicity, OPs are rapidly absorbed through mucosal surfaces and skin, and can even cross the blood–brain barrier to induce severe neurotoxicity [3]. The mechanism of organophosphorus poisoning involves the irreversible binding of organophosphorus compounds to acetylcholinesterase at cholinergic synapses, thereby inhibiting the hydrolysis of acetylcholine and leading to its accumulation. This accumulation induces a series of symptoms, including muscarinic, nicotinic, and central nervous system effects. In severe cases, it can result in coma, respiratory failure, and even death [4]. Actually, prolonged exposure to these chemicals can trigger neuroinflammation, oxidative stress, and changes in neurotransmitter systems, resulting in cognitive deficits, neuropsychiatric disorders, and, potentially, neurodegeneration [5]. In contemporary China, acute organophosphorus pesticide poisoning accounts for 20% to 50% of all poisoning cases annually, posing a significant threat to the life and health of patients [6]. Notably, approximately one-quarter of occupational OP poisonings and over 70% of pesticide-related poisonings occur via dermal absorption [7], underscoring an urgent need for targeted transdermal antidotes.

To meet this need, organophosphorus hydrolase (OPH)—a homodimeric protein isolated from *Pseudomonas* sp.—recognizes the specific chemical structure of organophosphorus compounds and binds to the OP substrate through amino acid residues in its active center. This initiates a hydrolysis reaction that cleaves the phosphorus-ester or phosphorus-sulfur bonds in OPs, converting toxic organophosphorus compounds into relatively non-toxic or low-toxicity products [8,9,10]. OPH is therefore recognized as a promising candidate for both prophylactic and therapeutic interventions against OP poisoning [11]. In recent years, directed evolution has successfully generated several OPH mutants with enhanced catalytic activity, including OPHDS5 (K185R/R319S), YT (H257Y/L303T), and G5C23 (K77A/A80V/F132E/T173N/H254G/I274N) [12,13,14,15,16,17]. However, in vivo delivery strategies for OPH remain underdeveloped. Although intravenous injection is currently the primary route of administration, it suffers from limitations such as procedural complexity, poor patient compliance, and increased medical waste [18]. Oral administration also faces challenges, as OPH is readily degraded by gastrointestinal enzymes, resulting in low bioavailability [19]. However, organophosphorus hydrolase may cause certain immunogenicity and instability, posing certain application challenges [20]. Therefore, alternative delivery approaches are urgently needed to overcome these clinical limitations.

To address the limitations of intravenous injection and oral delivery of (OPH), we attempt to use OPH-loaded microneedles for effective prevention and treatment of organophosphorus poisoning. Microneedles have emerged as a novel transdermal delivery platform capable of penetrating the stratum corneum barrier and delivering drugs directly into the dermal or lymphatic compartments, thereby facilitating rapid drug transport and absorption. Their minimally invasive nature, high efficiency, and ease of use have garnered widespread acceptance [21]. Dissolving microneedles are the most commonly used type, which are usually fabricated from synthetic polymers such as polyvinylpyrrolidone (PVP) and polyvinyl alcohol (PVA) [22]. However, polymer-based matrices have several drawbacks, including slow drug release that fails to meet the requirements for rapid-onset therapeutics, and high viscosity that complicates manufacturing [23,24]. Sulfobutylether-β-cyclodextrin (SCD), a water-soluble cyclodextrin derivative, has been reported to exhibit excellent biosafety and can form supramolecular cross-linked networks via hydrogen bonding and hydrophobic interactions in solution. Wang H. et al. prepared SCD-based microneedles with a dissolution time of less than 30 s. Loading with IBF achieved rapid onset of action and effective anti-inflammatory effects [25]. Lyu Wenchang constructed soluble supramolecular nanomicroneedles with excellent mechanical properties using SCD and UCNPs@PEI-RB@PEG-HKN15, which feature active targeting and NIR light responsiveness. These microneedles enabled spatiotemporally oriented induction of endogenous ferroptosis in keloids, achieving precise and intelligent keloid therapy and expanding the diagnostic and treatment approaches for keloids [26]. Wang H et al. developed a dissolving microneedle patch composed of SCD/tetramethylpyrazine (TPyP) supramolecular photosensitizers (PS) to enhance biofilm penetration and eradication. The supramolecular dissolving microneedles provide a promising platform for efficient biofilm removal and other photodynamic therapy (PDT) applications [27]. These unique properties endow SCD-based microneedles with robust mechanical strength and rapid dissolution capability, making them particularly suitable for encapsulating bioactive protein macromolecules [28,29].

To overcome the limitations of intravenous and oral delivery of organophosphorus hydrolase, this study for the first time loaded OPHDS5 into SCD microneedles. Upon administration, the microneedles instantly dissolve upon contact with interstitial fluid, rapidly releasing OPHDS5, which provides both preventive treatment and rapid rescue of SD rats from ethyl paraoxon poisoning.

## 2. Materials and Methods

### 2.1. Main Reagents and Instruments

Sulfobutylether-β-cyclodextrin (Cat. No. DL-132) was obtained from Xi’an Deli Biological Chemical Co., Ltd. (Xi’an, China). Trehalose (Cat. No. S11052-100 g) and mannitol (Cat. No. S11072-500 g) were purchased from Shanghai Yuanye Biotechnology Co., Ltd. (Shanghai, China). The BCA protein assay kit (Cat. No. 23227) was from Thermo Fisher Scientific (Waltham, MA, USA). Ethyl paraoxon (Cat. No. 311-45-5) was supplied by Beijing Wanjia Shouhua Biotechnology Co., Ltd. (Beijing, China). Trichloroacetic acid (Cat. No. T104257-500 g) was obtained from Shanghai Aladdin Biochemical Technology Co., Ltd. (Shanghai, China). Saline (Cat. No. G4702-500 mL) was from Servicebio Technology Co., Ltd. (Wuhan, China). FITC-labeled OVA protein solution (Cat. No. SF069) was purchased from Beijing Solarbio Science & Technology Co., Ltd. (Beijing, China). The microneedle mold (Cat. No. KX01) was from Henan Weina Benteng Biotechnology Co., Ltd. (Xinxiang, China). Trypan blue solution (0.4%, Cat. No. R20306) was from Shanghai Yuanye Biotechnology Co., Ltd. (Shanghai, China). HEPES buffer (pH 8.0, Cat. No. XBPY) was purchased from Shanghai Macklin Biochemical Co., Ltd. (Shanghai, China). Isoflurane (Cat. No. R510-22-10) was from Shenzhen RWD Life Science Co., Ltd. (Shenzhen, China). The Cy5.5 fluorescent labeling kit for antibodies/proteins (100 mg labeling scale, Cat. No. R-SJH-003) was from Xi’an Ruixi Biological Technology Co., Ltd. (Xi’an, China). Multifunction microplate reader (CLARIOstar) (VANTAstar FAL, BMG LABTECH, Offenburg, Germany); Biochemical incubator (LRH250, Shanghai Yiheng Scientific Instrument Co., Ltd., Shanghai, China); Single-hole water bath (HH-1, Changzhou Guohua Electric Appliances Co., Ltd., Changzhou, China); Freeze dryer (LGJ-25G, Beijing Sihuan Scientific Instrument Factory, Beijing, China); Protein purification system (Hass Device Run v3.0) (Hass25-M, Huiyin Technology (Beijing) Co., Ltd., Beijing, China); Zeiss inverted fluorescence microscope (labscope4) (Axiovert 5, Carl Zeiss AG, Oberkochen, Germany); Confocal laser scanning microscope (ZEN2 blue edition) (LSM 900, Carl Zeiss AG, Oberkochen, Germany); Optical microscope (v1.2.5) (XD30, Ningbo Sunny Instruments Co., Ltd., Ningbo, China); Scanning electron microscope (smartSEM 5.07) (GeminiSEM 300, Carl Zeiss AG, Oberkochen, Germany); Universal tensile tester (v4.10) (C42, MTS Systems (China) Co., Ltd., Shanghai, China); Refrigerated centrifuge (Centrifuge 5804R, Eppendorf (Shanghai) International Trade Co., Ltd., Shanghai, China); Small animal in vivo imaging system (AniView-V1.00.08360) (BLT aniview600, Guangzhou Biolight Biotechnology Co., Ltd., Guangzhou, China); Small animal anesthesia machine (R500I, Shenzhen RWD Life Science Technology Co., Ltd., Shenzhen, China); Transdermal diffusion tester (TP-6, Shanghai Yiheng Scientific Instrument Co., Ltd., Shanghai, China); High-speed benchtop centrifuge (GT318C, Hunan Kecheng Instrument Equipment Co., Ltd., Changsha, China).

### 2.2. Experimental Animals

Male specific pathogen-free (SPF) SD rats (3–8 weeks old, 180–200 g) and male SPF C57BL/6N mice (6–8 weeks old, approximately 20 g) were obtained from Sibelfu (Beijing) Biotechnology Co., Ltd. (Beijing, China) under license SCXK (Jing) 2024-001. In the in vivo imaging system (IVIS) characterization, we used C57BL/6N mice primarily because of their small body size, which is more suitable for long-term monitoring of whole-body fluorescence distribution within the field of view of our small animal live imaging system. Sprague-Dawley rats were used for pharmacokinetics, pharmacodynamics, and safety assessments because they have larger blood volume for serial sampling, and their skin structure and toxicological responses are more relevant for evaluating organophosphate exposure. Animals were housed at 22–25 °C with 45–55% relative humidity under a 12 h light/dark cycle (lights on 06:00–21:00). Food and water were provided ad libitum, and bedding was changed every 3 days. All animal procedures were approved by the Experimental Animal Ethics Committee of the Experimental Animal Center, Academy of Military Medical Sciences (Approval No. DWZX-IACUC-2026-511).

### 2.3. Preparation of Solutions

#### 2.3.1. Preparation of Needle Tip Solution

To ensure the fast-dissolving property of the microneedles, we selected 60% *w*/*v* SCD as the matrix solution. Lyophilized OPHDS5 powder (60 mg) was reconstituted with 2.08 mL of distilled water in a 50 mL Eppendorf tube, followed by vortexing. SCD (1.248 g) was subsequently added to the tube and vortexed to prepare a 60% *w*/*v* OPHDS5-loaded SCD solution.

#### 2.3.2. Preparation of Backing Layer Solution

To provide adequate mechanical support, we selected 150% *w*/*v* SCD solution as the backing layer of the microneedles. 150% *w*/*v* SCD solution was prepared by accurately weighing 6.0 g of SCD into a 50 mL Eppendorf tube, adding 4 mL of distilled water, and vortexing the mixture until homogeneous.

### 2.4. Screening of Matrix Materials for Blank Microneedles

In this study, various matrix materials were screened to identify a suitable candidate for microneedle fabrication that combines rapid dissolution with favorable mechanical properties. The polydimethylsiloxane (PDMS) mold used for microneedle fabrication contained a 15 × 15 array of square-pyramidal cavities, each with a length of 1540 μm and a base width of 600 μm. Solutions of maltose (20, 40, 60, 80, 100% *w*/*v*), dextran 20 (10, 15, 20% *w*/*v*), trehalose (6, 8, 10% *w*/*v*), and SCD (60, 90, 120, 150, 180% *w*/*v*) were prepared. The viscosity of each solution was measured [28]. Maltose was weighed at 1.0, 2.0, 3.0, 4.0, and 5.0 g, respectively; dextran 20 was weighed at 0.5, 0.75, and 1.0 g, respectively; trehalose was weighed at 0.3, 0.4, and 0.5 g, respectively; and SCD was weighed at 2.0, 4.5, 6.0, 7.5, and 9.0 g, respectively. Each was diluted with deionized water to a final volume of 5 mL. After vortexing, 550 μL of each solution was transferred and centrifuged at 3000 r/min for 10 min [30] to prepare microneedles. The ease of demolding and the needle morphology after demolding were observed.

### 2.5. Preparation of OPHDS5-Loaded Microneedles

**Figure 1:**
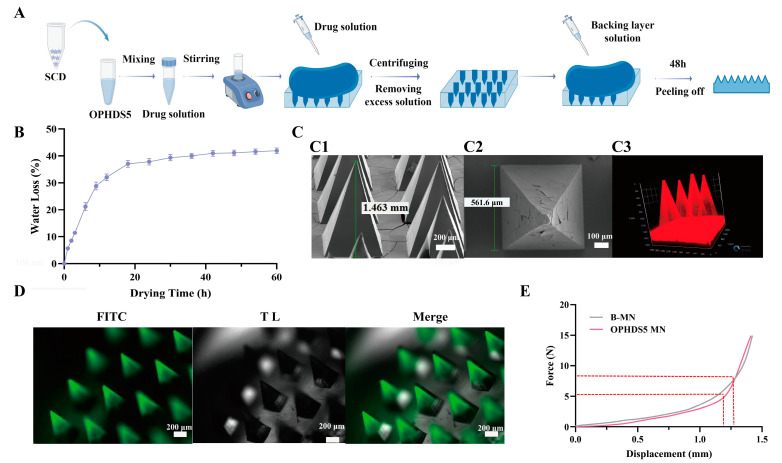
Fabrication and characterization of OPHDS5-loaded microneedles. (**A**) Schematic of the fabrication process. (**B**) Water loss rate versus drying time for microneedles (mean ± SD, n = 3). (**C**) Representative images of OPHDS5-loaded microneedles: (C1) SEM front view (scale bar: 100 μm); (C2) SEM top view (scale bar: 200 μm); (C3) CLSM image showing OPHDS5 distribution. (**D**) Fluorescence microscopy image of FITC-OVA distribution in microneedle tips (scale bar: 200 μm). In the figure, green indicates FITC fluorescence staining, and gray indicates the bright-field image. (**E**) Force-displacement curves for a single blank microneedle and an OPHDS5-loaded microneedle.

We fabricated OPHDS5-loaded microneedles using a centrifugal casting method; the fabrication process is illustrated in Figure 1A. Briefly, 550 μL of OPHDS5-loaded SCD solution was dispensed into the PDMS microneedle mold and centrifuged at 3000 rpm for 10 min to concentrate the drug into the needle tips. Excess solution outside the cavities was removed, and 500 μL of 150% *w*/*v* SCD solution was added as the backing layer. The mold was placed in a desiccator at room temperature for 48 h, after which the microneedles were demolded to obtain the drug-loaded patch.

To determine the optimal demolding time, a gravimetric method was employed [31]. The molds containing the drug and backing solutions were placed in a desiccator and weighed at various time intervals. The water loss rate was calculated as: Water loss (%) = (Initial weight M_0_—Weight after drying M)/Initial weight M_0_ × 100%. A curve of water loss versus drying time was plotted.

### 2.6. Characterization of Microneedles

#### 2.6.1. Morphological Characterization

To clearly observe the morphological characteristics of the microneedles and the distribution of OPHDS5 within them, we characterized the enzyme-loaded microneedles using scanning electron microscopy (SEM) (Carl Zeiss AG, Oberkochen, Germany) [32], fluorescence microscopy (Carl Zeiss AG, Oberkochen, Germany) [33] and confocal laser scanning microscopy (CLSM) (Carl Zeiss AG, Oberkochen, Germany) [34]. Microneedles were sputter-coated with gold and imaged with a GeminiSEM 300 (Carl Zeiss AG, Oberkochen, Germany) to obtain top and side views of the 15 × 15 array. Using FITC-labeled OVA as a model protein, the distribution of the protein on the microneedle shafts was observed using an inverted fluorescence microscope (Axiovert 5) (Carl Zeiss AG, Oberkochen, Germany). The FITC-labeled OVA solution was prepared as follows: 1.2 mg of SCD was added to 2 mL of FITC-labeled OVA solution, vortexed to mix thoroughly, and then microneedles were prepared following the method described in Section 2.4.

Cy5.5-labeled OPHDS5 was prepared as follows: 5 mL of OPHDS5 solution (2 mg/mL) was mixed with 0.1 mL of Coupling Reagent. Separately, 1 mg of Cy5.5-mix solid was dissolved in 0.2 mL of Cy5.5-mix solvent and added to the OPHDS5 solution. The mixture was incubated for 30 min at room temperature in the dark, then transferred to an ultrafiltration tube and centrifuged at 10,000× *g* for 5 min to remove unbound Cy5.5-mix. The retained protein was resuspended in distilled water to obtain Cy5.5-labeled OPHDS5. The protein concentration was determined using a BCA assay (Thermo Fisher Scientific, Waltham, MA, USA) and diluted to 5.8 mg/mL. One milliliter of this solution was mixed with 0.6 g of accurately weighed SCD, vortexed, and used to fabricate Cy5.5-labeled OPHDS5 microneedles by centrifugation as described in Section 2.4. The distribution of labeled OPHDS5 within the microneedles was imaged using an LSM 900 CLSM (Carl Zeiss AG, Oberkochen, Germany).

#### 2.6.2. Mechanical Property Evaluation

To identify the SCD concentration that yields optimal mechanical properties, microneedles prepared with various SCD concentrations were tested. The microneedle patch was flattened and attached to a fixed plate with double-sided tape. A single compression test was performed using a universal tensile tester (MTS Systems (China) Co., Ltd., Shanghai, China) with a maximum force of 150 N and a probe descending speed of 1 mm/s. Force-displacement curves within 800 μm were recorded [35] to compare the mechanical performance of microneedles with different SCD concentrations.

The mechanical properties of the OPHDS5-loaded and blank microneedle patches were both evaluated. The patches were cut into 1 × 10 arrays using a scalpel. Force-displacement curves per needle were generated to compare the mechanical properties after enzyme loading.

#### 2.6.3. Evaluation of Microneedle Dissolution In Vitro and In Vivo

To evaluate whether the microneedles achieved a fast-dissolving effect, this study investigated their dissolution rate both in vitro and in vivo. The in vitro dissolution time of microneedle tips prepared with different SCD concentrations (60, 90, 120, 150, 180% *w*/*v*) was assessed. The backing layer of a microneedle patch was held with forceps, and the tips were gently touched to the water surface in a Petri dish at room temperature. The time required for the complete dissolution of the needle tips was recorded (n = 3).

Male SD rats were anesthetized with isoflurane using an anesthesia machine (n = 3). After shaving the dorsal skin, microneedle patches were inserted perpendicularly into the skin and pressed with the thumb for 30 s. The patches were then removed at 0, 0.5, 1, 2, and 5 min after the end of the pressing. Images of the microneedle shafts at different time points were captured under an optical microscope (Ningbo Sunny Instruments Co., Ltd., Ningbo, China) to observe the differences in dissolution morphology over time [36].

#### 2.6.4. Evaluation of Microneedle Skin Penetration Performance

To visualize skin penetration, microneedles were prepared as described in Figure 1, except that the water was replaced with a 0.4% *w*/*v* trypan blue solution. Briefly, 36 mg of lyophilized OPHDS5 was placed in a 50 mL EP tube, and 1.25 mL of 0.4% Trypan Blue solution was added and vortexed. Accurately weighed 0.75 g of SCD was added, vortexed, and used to fabricate microneedles. After demolding, the microneedles were applied to the shaved dorsal skin of SD rats; after 5 min, the microneedles were removed, and the presence of blue punctate arrays was examined [37].

At the same time, we also evaluated the piercing performance of the microneedles using Parafilm. The Parafilm was placed on the surface of a sponge, and the microneedles were inserted vertically into the Parafilm with an applied force. After 5 min, the microneedles were removed, and the puncture sites were observed.

The penetration depth of the microneedles was further evaluated by hematoxylin and eosin (H&E) staining [38]. Microneedles were applied to the shaved dorsal skin of SD rats and pressed for 5 min. The treated skin area (approximately 2 cm × 2 cm × 0.3 cm) was excised and fixed in 4% *w*/*v* paraformaldehyde at room temperature for 24 h. The tissue was then dehydrated, embedded in paraffin, sectioned, and stained with H&E. Sections were examined under a light microscope (Ningbo Sunny Instruments Co., Ltd., Ningbo, China) to measure the depth and width of microneedle insertion.

#### 2.6.5. Evaluation of In Vitro Transdermal Release

To evaluate drug loading and release kinetics, an in vitro release study was conducted. SD rats were euthanized after dorsal hair removal, and the dorsal skin was excised, cleaned of subcutaneous fat, and rinsed with saline (n = 3). After air-drying, a microneedle patch was inserted into the skin with thumb pressure for 30 s, and the skin was mounted between the donor and receiver compartments of a Franz diffusion cell with the microneedle backing facing the donor compartment [39]. The receiver compartment contained 17.4 mL of PBS (pH 7.4) to maintain sink conditions. The effective diffusion area was 2.54 cm^2^, and the system was maintained at 37 °C with magnetic stirring at 150 rpm. At predetermined time points (2, 5, 10, 20, 40, 60, 80, 100, 120 min), 0.5 mL samples were withdrawn and replaced with an equal volume of fresh PBS. Samples were filtered through a 0.22 μm membrane, and the OPHDS5 content was determined using a BCA protein assay kit (Thermo Fisher Scientific, Waltham, MA, USA). The cumulative drug permeation amount was calculated using the following formula:

$$Q_{n}=C_{n}V+\sum_{i=1}^{n-1}C_{i}V_{i}$$

where *Q_n_* is the cumulative drug permeation amount at the nth sampling time point (μg), *C_n_* is the drug concentration in the nth sample (μg·mL^−1^), *V* is the volume of the diffusion cell (17.4 mL), and *V_i_* is the sampling volume (0.5 mL).

#### 2.6.6. In Vivo Imaging System (IVIS) Characterization

The systemic distribution of OPHDS5 following microneedle administration was tracked using small animal in vivo imaging technology. Cy5.5-labeled OPHDS5 microneedles were prepared as described in Section 2.6.1, considering the body weight difference between mice (~20 g) and SD rats (~200 g). The microneedle patch was cut into two equal pieces, and one piece was applied to each mouse (n = 3). After hair removal, the microneedle patch was applied to the dorsal skin of the mice and removed 5 min later. At predetermined time points (0, 2, 4, 8, 16, 24, 32, 40, 48, and 72 h), the mice were imaged using an in vivo imaging system to observe the biodistribution of fluorescence and the relative fluorescence intensity between the administration site and the whole body [40].

### 2.7. Pharmacokinetic Evaluation

To verify the distribution of OPHDS5 observed in in vivo imaging, this study determined the plasma concentration of OPHDS5 using an indirect method. Using ethyl paraoxon as the substrate, OPHDS5 activity in plasma samples collected at various time points was measured to indirectly determine plasma OPHDS5 concentration, and an activity-concentration curve was generated [41].

Eighteen male SD rats were divided into three groups (n = 6 per group): subcutaneous injection, intramuscular injection, and OPHDS5-loaded microneedle patch. After collecting blank plasma, rats in the subcutaneous and intramuscular groups received 1.2 mg of OPHDS5 solution. Rats were anesthetized with isoflurane (3%), and blood samples (100 μL) were collected from the orbital plexus at 5, 15, and 30 min, and 1, 3, 6, 12, 24, 48, and 72 h post-administration. For the microneedle group, patches were prepared as described in Section 2.4, applied to the shaved dorsal skin with thumb pressure for 5 min, and secured with medical tape. Blood samples (100 μL) were collected at 0.5, 1, 2, 4, 8, 16, 24, 32, 40, 48, and 72 h into heparinized tubes, centrifuged at 2500× *g* for 10 min, and the plasma was stored at −80 °C. All plasma samples were diluted 10-fold with 50 mM HEPES buffer (pH 8.0), and OPHDS5 activity was measured by monitoring absorbance at 405 nm. The OPHDS5 concentration in plasma was calculated, and the concentration-time curve was plotted.

### 2.8. Evaluation of the Prophylactic Effect of OPHDS5-Loaded Microneedles Against Ethyl Paraoxon Poisoning in SD Rats

#### 2.8.1. In Vitro Enzyme Activity of OPHDS5-Loaded Microneedles

To verify the enzymatic activity of OPHDS5 within the microneedles, an in vitro enzyme activity assay was performed prior to the animal experiments. Ten 5 mL centrifuge tubes were numbered 1–10. Varying volumes of 6 mM p-nitrophenol solution, 50 mM HEPES buffer (pH 8.0), 10% *w*/*v* trichloroacetic acid, and 10% *w*/*v* sodium carbonate were added to generate a standard curve of absorbance at 405 nm versus p-nitrophenol concentration. OPHDS5-loaded microneedles were dissolved in distilled water, and the volume was adjusted to 1 mL in a volumetric flask. The protein concentration was determined using a BCA assay, and the solution was diluted to 500 ng/mL. Enzyme activity before and after microneedle fabrication was measured and compared.

#### 2.8.2. Evaluation of Prophylactic Efficacy Against Ethyl Paraoxon Poisoning

To evaluate the prophylactic efficacy of OPHDS5-loaded microneedles against ethyl paraoxon poisoning, the corresponding pharmacodynamic study was subsequently conducted. Twenty-four SD rats were divided into four groups (n = 6 per group): blank microneedle group, low-dose (0.3 mg OPHDS5), medium-dose (0.6 mg OPHDS5), and high-dose (1.2 mg OPHDS5) microneedle groups. Based on our preliminary experiments [41], rats received a subcutaneous injection of 2× LD_50_ (0.658 mg/kg) ethyl paraoxon at the microneedle application site 8 h after microneedle administration. Microneedles were applied with thumb pressure for 5 min and secured with medical tape. After 8 h, the tape was removed, and rats were challenged subcutaneously. The survival status of rats was observed within 30 min, and the body weight and temperature changes in the surviving rats were recorded over the 6 days following the challenge.

#### 2.8.3. Evaluation of First-Aid Efficacy Against Ethyl Paraoxon Poisoning

To evaluate the rescue efficacy of OPHDS5-loaded microneedles against ethyl paraoxon poisoning, the corresponding pharmacodynamic study was conducted. Twenty-four SD rats were divided into four groups: Control, Untreated (ethyl paraoxon only), Blank MN (ethyl paraoxon + blank microneedles), and OPHDS5 MN (ethyl paraoxon + OPHDS5-loaded microneedles). The dorsal hair was shaved, and a 1 cm × 1 cm area was marked. A dose of 2× LD_50_ ethyl paraoxon was applied topically to this area, and the methanol solvent was allowed to evaporate. After 5 min, the Untreated group received no further intervention, the Blank MN group received a blank microneedle patch, and the OPHDS5 MN group received an OPHDS5-loaded microneedle patch. Poisoning symptoms were observed for 20 min post-exposure. An open field test was conducted during a 20–30 min interval to assess locomotor activity via movement trajectories. The body weight and temperature changes in the surviving rats were recorded over the 6 days following the challenge.

### 2.9. Safety Evaluation of OPHDS5-Loaded Microneedles

Finally, routine blood tests, serum biochemical analyses, and H&E staining were performed in this study to evaluate the safety of the OPHDS5-loaded microneedles. Six male SD rats with shaved dorsal hair were divided into a healthy control group and a microneedle patch group (n = 3 per group). Microneedles were applied to the dorsal skin with thumb pressure for 5 min and secured with medical tape; the tape was removed after 8 h. The control group received no treatment. To assess potential toxicity, rats were anesthetized with isoflurane 7 days later, and blood samples (2 mL each) were collected from the abdominal aorta into tubes for routine blood analysis and serum biochemistry. Routine blood parameters were measured immediately, and serum was obtained by centrifugation for biochemical analysis.

Three rats from each group were euthanized, and the heart, liver, spleen, lungs, kidneys, and dorsal skin were collected. Tissues were processed for H&E staining to examine histopathological changes 7 days after administration [30].

### 2.10. Statistical Analysis

Statistical analysis and graphing were performed using GraphPad Prism 10.0 software. Data are presented as mean ± standard deviation (mean ± SD). Comparisons among multiple groups were conducted using one-way ANOVA, and pairwise comparisons were performed using Student’s *t*-test. A *p*-value < 0.05 was considered statistically significant.

## 3. Results

### 3.1. Preparation and Characterization of Microneedles

#### 3.1.1. Morphological Characterization of Microneedles

The microneedle fabrication results using different matrix materials are shown in Figure S1. Microneedles prepared with trehalose, dextran 20, or maltose were difficult to demold and exhibited a flaky morphology, whereas those fabricated with SCD displayed regular square-pyramidal tips and were easily demolded. The mechanical strength of microneedles prepared with different concentrations of SCD was subsequently evaluated. As shown in Figure S2, all SCD-formulated microneedles exhibited favorable mechanical properties, with no fracture observed during testing. Notably, microneedles fabricated with 150% *w*/*v* SCD withstood the highest force at the same displacement without breaking; therefore, this concentration was selected for the backing layer. Meanwhile, 60% *w*/*v* SCD, which displayed the lowest viscosity (Table S1) and ease of preparation, was chosen as the matrix material for the needle tips. Therefore, the fabrication process for the microneedles is illustrated in Figure 1A. Briefly, SCD was added to the OPHDS5 solution to prepare a 60% *w*/*v* SCD solution containing OPHDS5. After vortexing, the mixture was dispensed into a PDMS mold and centrifuged for 10 min. The excess backing layer solution was then removed, and a 150% *w*/*v* SCD solution was added to form the backing layer. Following drying for 48 h, the resulting OPHDS5-loaded microneedles were demolded.

The water loss curve (Figure 1B) plateaued between 40 and 60 h; thus, 48 h was chosen as the optimal drying time. OPHDS5-loaded fast-dissolving microneedles were successfully fabricated using the PDMS mold and centrifugation method. Representative SEM, fluorescence microscopy, and CLSM images are shown in Figure 1C,D. Scanning electron microscopy images show that the demolded microneedles have a length of 1.463 mm and a base width of 561.6 μm; the needle shafts are regular square pyramids, neatly arranged in a 15 × 15 array, with no obvious bubbles. No bubbles or cavities were observed. Fluorescence microscopy revealed uniform distribution of the model protein (FITC-labeled ovalbumin) within the needle tips, indicating good compatibility between the protein and SCD matrix. In Figure 1C, red fluorescence appears in the backing layer, whereas in Figure 1D, fluorescence is distributed only in the microneedle tips. The analysis is as follows: Residual excess Cy5.5-labeled OPHDS5 solution remained in the backing layer and was not completely removed. The imaging principles of confocal laser scanning microscopy and fluorescence microscopy are different. Confocal laser scanning microscopy was used to directly observe the three-dimensional distribution of OPHDS5 inside the microneedles, and the results showed that OPHDS5 was uniformly distributed throughout the entire microneedle structure. Using FITC-labeled OVA as a model drug, fluorescence microscopy was employed to verify the tip-loading process, capturing only the fluorescence projection on the surface. The results indicated that microneedles prepared by the centrifugation method mainly concentrated the model drug in the microneedle tips, which meets the design requirements for transdermal drug delivery. Both types of microneedles were prepared using the same fabrication method, and subsequent pharmacodynamic evaluations have confirmed that this does not affect drug delivery efficiency.

#### 3.1.2. Mechanical Property Evaluation

As shown in Figure 1E, blank microneedles reached a maximum force of approximately 7.5 N per needle at 1.3 mm displacement, while OPHDS5-loaded microneedles deformed at 1.2 mm with a force of approximately 4 N per needle. Although slightly lower than blank microneedles, this force exceeded the reported minimum force required for stratum corneum penetration (0.38 N per needle) [42], confirming that the loaded microneedles meet the mechanical requirements for effective skin insertion.

#### 3.1.3. Skin Penetration Performance of Microneedles

**Figure 2:**
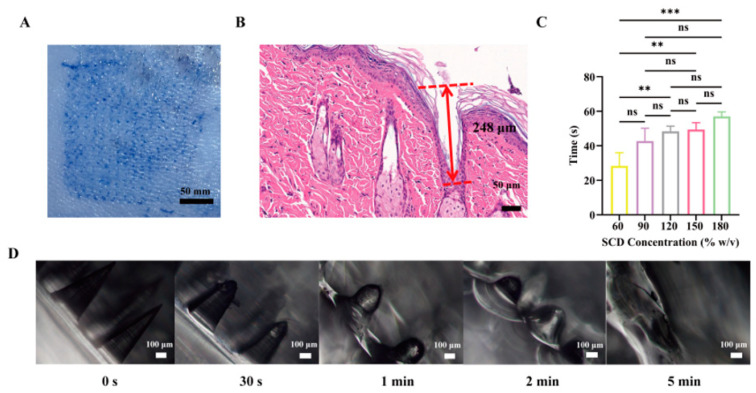
Characterization of OPHDS5-loaded microneedles after administration in vitro and in vivo. (**A**) Image of excised rat skin stained with 0.4% Trypan Blue after microneedle application, showing blue puncture marks. (**B**) H&E-stained section of rat skin after microneedle insertion (scale bar: 50 μm). (**C**) Dissolution time of microneedles prepared with various SCD concentrations (mean ± SD, n = 3). A statistically significant difference was observed compared to the 60% *w*/*v* SCD group (ns *p* > 0.05, ** *p* < 0.01, *** *p* < 0.001). (**D**) Optical microscopy images of microneedle tips after application to rat skin for different durations (scale bar: 100 μm).

As shown in Figure 2A, Trypan Blue staining revealed distinct blue microchannels on the excised skin, corresponding to microneedle puncture sites arranged in a neat array. Supplementary Figure S3 shows that fewer than 10 individual needles failed to penetrate the semipermeable membrane, achieving a penetration rate of over 95% and leaving clearly visible pores. H&E staining of rat dorsal skin (Figure 2B) demonstrated that microneedles penetrated to a depth of approximately 248 μm, skin indentation and the relatively wide base of the microneedles limit full insertion, and the sectioning and embedding processes during H&E staining lead to an underestimation of the measured depth [43]. Although only the needle tips penetrate into the skin, most of the active enzyme is successfully delivered to the dermis, still confirming excellent skin insertion capability.

#### 3.1.4. Dissolution of Microneedles In Vitro and In Vivo

All microneedles prepared with different SCD concentrations dissolved completely within 60 s in vitro, indicating good dissolution properties (Figure 2C). Microneedles fabricated with 60% *w*/*v* SCD exhibited the fastest dissolution, with tips dissolving within approximately 30 s. Therefore, 60% *w*/*v* SCD was selected as the matrix material for subsequent studies. Regarding vivo dissolution assessment, Figure 2D shows that microneedle tips began to dissolve within 30 s of application, most of the needle body dissolved by 1 min, and nearly complete dissolution was observed by 5 min, confirming the fast-dissolving nature of the microneedles.

#### 3.1.5. In Vitro Transdermal Release of Microneedles

**Figure 3:**
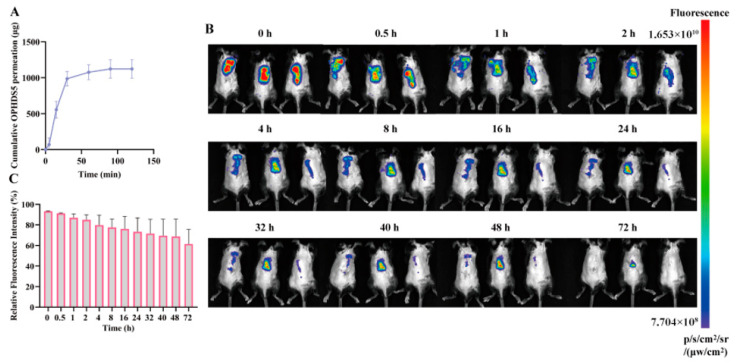
In vitro release and in vivo imaging of OPHDS5-loaded microneedles. (**A**) Cumulative in vitro transdermal release profile of OPHDS5 (n = 3). (**B**) Representative in vivo fluorescence images of mice at various time points after application of Cy5.5-labeled OPHDS5 microneedles (n = 3). (**C**) Percentage of fluorescence intensity remaining at the application site relative to total body fluorescence over time (mean ± SD, n = 3). No significant differences were observed among time points (*p* > 0.05).

The cumulative permeation profile of OPHDS5 exhibited a typical biphasic release pattern (Figure 3A). During the initial phase (0–60 min), a burst release was observed, with the cumulative amount increasing linearly to approximately 980 μg, attributed to rapid diffusion of OPHDS5 from the surface of the microneedles. In the second phase (60–120 min), the permeation rate declined, and the cumulative amount plateaued at approximately 1080 μg, reflecting sustained release of the remaining drug.

#### 3.1.6. In Vivo Imaging of Cy5.5-Labeled OPHDS5 Microneedles

Following application of Cy5.5-labeled OPHDS5 microneedles to the dorsal skin, fluorescence was monitored at various time points to track drug biodistribution and clearance (Figure 3B,C). In the early phase (0–4 h), intense fluorescence was localized at the application site, with nearly 100% of the signal confined to the local area, indicating rapid drug release from the microneedles and minimal systemic diffusion. During the intermediate phase (8–24 h), fluorescence intensity gradually decreased but remained high, with 70–90% of the signal still localized, suggesting sustained drug retention in the skin. In the late phase (32–72 h), fluorescence further diminished, and the local signal fraction dropped to approximately 60%, reflecting gradual drug metabolism and clearance. These findings demonstrate prolonged local retention of the macromolecular protein after microneedle administration.

### 3.2. Pharmacokinetic Evaluation of OPHDS5-Loaded Microneedles

**Figure 4:**
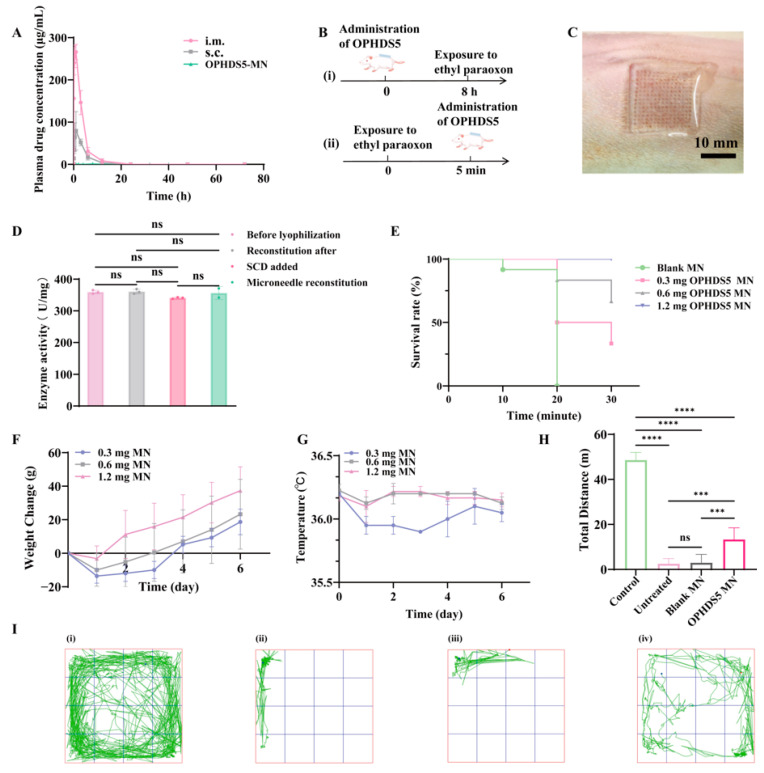
Prophylactic and first-aid efficacy of OPHDS5-loaded microneedles against ethyl paraoxon poisoning in SD rats. (**A**) Plasma concentration–time profiles of OPHDS5 after intramuscular injection, subcutaneous injection, and microneedle administration (mean ± SD, n = 6). (**B**) (i) and (ii) are the flowcharts of the prevention experiment and the antidote experiment against ethyl paraoxon poisoning, respectively. (**C**) Photograph of a microneedle patch applied to rat skin. (**D**) Enzyme activity of OPHDS5 before and after lyophilization, after mixing with SCD, and after microneedle fabrication (mean ± SD, n = 3). ns *p* > 0.05. (**E**) Survival curves of rats in blank microneedle and various OPHDS5 dose groups following ethyl paraoxon challenge (n = 6). (**F**) Body weight changes over 6 days post-challenge (n = 6). (**G**) Body temperature changes over 6 days post-challenge (n = 6). (**H**) Total distance traveled in an open field test during the 20–30 min interval post-challenge (mean ± SD, n = 6). (**I**) Representative movement trajectories of rats from each group. (i)–(iv) show representative images of the movement trajectories of rats in the Control, Untreated, Blank MN, and OPHDS5 MN groups, respectively. In the images, the black and red dots represent the start and end points of rat activity, respectively, and the green lines represent the movement trajectories of the rats. ns *p* > 0.05, *** *p* < 0.001, **** *p* < 0.0001.

Pharmacokinetic profiles are shown in Figure 4A. Intramuscular injection of OPHDS5 resulted in a rapid peak plasma concentration of approximately 300 μg/mL, followed by a sharp decline to near-zero levels by 20 h. Subcutaneous injection produced a lower peak (~100 μg/mL) with a similar rapid rise and fall, albeit with a slower absorption rate. Both injection routes led to rapid systemic exposure without sustained release. In contrast, plasma OPHDS5 concentrations following microneedle administration remained consistently low (near 0 μg/mL) throughout the 72 h study period, with no discernible peak. This indicates that microneedle delivery achieves localized drug retention with minimal systemic exposure, thereby avoiding potential side effects associated with systemic circulation.

### 3.3. Pharmacodynamic Evaluation of OPHDS5-Loaded Microneedles

#### 3.3.1. Prophylactic Efficacy of OPHDS5-Loaded Microneedles Against Ethyl Paraoxon Poisoning

The experimental design for assessing the prophylactic effect is illustrated in Figure 4B(i). Briefly, microneedles were injected perpendicularly into the depilated dorsal skin of rats. (Figure 4C). Eight hours post-administration, ethyl paraoxon was injected subcutaneously at the same site, and the outcomes of the prophylactic study were observed.

As shown in Figure 4D, in vitro enzyme activity assays confirmed that SCD, the lyophilization process, and the microneedle fabrication procedure did not compromise OPHDS5 activity, with normal enzyme function retained throughout. Based on pharmacokinetic findings suggesting minimal systemic absorption following transdermal delivery of OPHDS5-MN, subsequent challenges were administered subcutaneously at the microneedle application site. Survival rate results (Figure 4E) demonstrated a significant dose-dependent increase in rat survival as the dose escalated from 0.3 mg to 1.2 mg, with the 1.2 mg dose group achieving 100% survival. This indicates that the protective effect is dose-dependent and that OPHDS5 administered via microneedles can effectively reach the skin and subcutaneous tissue to exert its protective role. Dynamic monitoring of body weight and temperature (Figure 4F,G) revealed that rats in the 0.3 mg low-dose group exhibited significant initial decreases in both parameters, followed by gradual recovery. In contrast, the medium- and high-dose groups showed smaller declines and faster recovery, with body weight trending upward, indicating better maintenance of physiological homeostasis and reduced pathophysiological damage from poisoning.

#### 3.3.2. Rescue Efficacy of OPHDS5-Loaded Microneedles Against Ethyl Paraoxon Poisoning

The experimental design for evaluating the first-aid efficacy of OPHDS5-loaded microneedles is illustrated in Figure 4B(ii). Following depilation, ethyl paraoxon was topically applied to the dorsal skin of SD rats. Five minutes post-exposure, microneedles were administered perpendicularly at the same site, and the detoxification effect was assessed using the open field test. As shown in Figure 4H,I, no significant difference in total travel distance was observed between the Untreated group and the Blank MN group after ethyl paraoxon challenge, indicating that blank microneedles neither caused significant damage nor exerted any therapeutic effect. In contrast, the OPHDS5 MN group exhibited significantly greater travel distance compared to these two groups, demonstrating that rats treated with OPHDS5-loaded microneedles retained a certain level of locomotor activity. Although their activity was lower than that of the Control group, it was notably higher than that of the other two groups, confirming the therapeutic efficacy of OPHDS5-loaded microneedles against percutaneous ethyl paraoxon poisoning. Supplementary Figures S4 and S5 illustrate that over the 6 days post-exposure, the OPHDS5 microneedle treatment group exhibited slightly higher body weight gain compared to the other two groups. The blank microneedle group displayed the lowest body weight and temperature, likely because disruption of the stratum corneum during its application facilitated faster percutaneous penetration of ethyl paraoxon. The differences in therapeutic efficacy among the three groups were primarily reflected in locomotor activity levels following ethyl paraoxon challenge, with overall differences in body weight and temperature being relatively minor.

### 3.4. Safety Evaluation of OPHDS5-Loaded Microneedles

**Figure 5:**
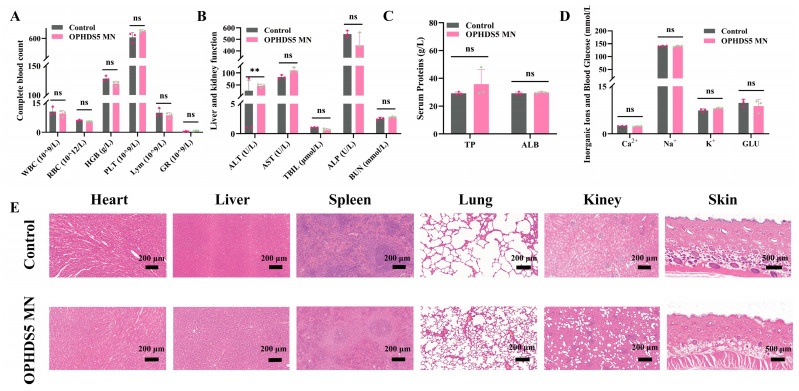
Safety evaluation of OPHDS5-loaded microneedles. (**A**) Routine hematological parameters in control and OPHDS5-MN treated rats 7 days post-application (mean ± SD, n = 3). The individual points above the bars denote the raw measurement values of each replicate sample. Pink dots represent the Control group, and green dots represent the OPHDS5 MN group. (*p* > 0.05). (**B**) Serum biochemical markers of liver and kidney function (mean ± SD, n = 3). (**C**) Serum protein levels (mean ± SD, n = 3). (**D**) Serum electrolyte and glucose levels (mean ± SD, n = 3). (**E**) Representative H&E-stained sections of major organs and skin from control and OPHDS5-MN groups (n = 3). Scale bar: 200 μm (Heart, Liver, Spleen, Lung, Kidney), 500 μm (Skin). ns *p* > 0.05, ** *p* < 0.01.

As shown in Figure 5A, no significant differences were observed between the control and OPHDS5-MN groups in key inflammatory and hematopoietic parameters, including white blood cell count (WBC), red blood cell count (RBC), hemoglobin (HGB), platelet count (PLT), lymphocytes (Lym), and granulocytes (GR), indicating that the treatment did not induce acute inflammation or hematopoietic disruption. Serum biochemical analysis (Figure 5B–D) revealed no significant differences in liver function markers (aspartate aminotransferase, AST; alkaline phosphatase, ALP; total bilirubin, TBIL), kidney function markers (blood urea nitrogen, BUN), serum proteins (total protein, TP; albumin, ALB), electrolytes (Na^+^, Ca^2+^, K^+^), or blood glucose (GLU), except for a slight fluctuation in alanine aminotransferase (ALT). These results suggest that OPHDS5-MN did not significantly impair hepatic or renal function or disrupt electrolyte and energy homeostasis. Histological examination of major organs (heart, liver, spleen, lungs, kidneys) and skin (Figure 5E) showed normal architecture with no evidence of inflammatory infiltration, cellular degeneration, or tissue abnormalities in either group. The histomorphological features were comparable between control and treated groups. Collectively, these findings demonstrate that OPHDS5-loaded microneedles are safe and do not cause significant physiological disturbances or tissue damage.

## 4. Discussion

In this study, we successfully fabricated fast-dissolving microneedles encapsulating OPHDS5 using SCD as the matrix material via a centrifugation method. The minimum prophylactic dose required to protect SD rats against lethal ethyl paraoxon poisoning was determined to be 1.2 mg, thereby establishing a novel minimally invasive strategy for the transdermal delivery of organophosphorus hydrolase and for the prevention and treatment of transdermal ethyl paraoxon poisoning.

The selection of SCD as the matrix material was pivotal to the success of this delivery system. As a cyclodextrin derivative, SCD possesses a unique amphiphilic cavity capable of accommodating hydrophobic domains of proteins, potentially stabilizing the enzyme during fabrication and storage [25]. Moreover, SCD forms supramolecular cross-linked networks through hydrogen bonding and hydrophobic interactions, which confer excellent moldability and mechanical strength to the microneedles. Compared to conventional polymers such as PVP or PVA, SCD solutions exhibit lower viscosity, facilitating mold filling and centrifugation, while the resulting microneedles display superior tip sharpness and uniform drug distribution [19]. Our comparative screening of various materials confirmed that SCD yielded the best needle morphology and demolding properties, underscoring its suitability for protein-loaded microneedle fabrication. The microneedles exhibited sufficient mechanical strength to penetrate the stratum corneum and create microchannels.

A key feature of the developed microneedles is their rapid dissolution, both in vitro (within 30 s) and in vivo (within 5 min). This rapid dissolution addresses the slow release limitation of conventional polymer-based microneedles, which often require minutes to hours to dissolve [25], thereby delaying drug absorption. In the context of organophosphorus poisoning, where rapid intervention is critical to prevent irreversible acetylcholinesterase inhibition and subsequent mortality, the fast-dissolving property of our microneedles is particularly advantageous. The biphasic release profile observed in vitro—an initial burst followed by sustained release—suggests that a fraction of OPHDS5 is rapidly available for immediate detoxification, while the remainder provides prolonged local protection.

In vivo imaging and pharmacokinetic studies revealed that OPHDS5 remained predominantly localized at the administration site, with minimal systemic absorption. This localized retention is highly desirable for treating percutaneous poisoning, as it concentrates the enzyme at the portal of entry, maximizing local hydrolysis of ethyl paraoxon while avoiding systemic side effects. OPHDS5 remained detectable in the skin for up to 72 h, suggesting that a single microneedle application can provide sustained protection against repeated or continuous dermal exposure. This contrasts sharply with intramuscular and subcutaneous injections, which resulted in rapid systemic absorption and clearance, offering no local depot effect. The pharmacokinetic profile of microneedle delivery thus represents a paradigm shift from systemic to local antidote administration, potentially reducing the required dose and minimizing off-target effects.

The prophylactic efficacy of OPHDS5-loaded microneedles was dose-dependent, with 1.2 mg providing complete protection against a 2 × LD_50_ challenge of ethyl paraoxon. This dose is considerably lower than that typically required for systemic administration, likely due to the localized delivery and sustained retention at the exposure site. The preservation of body weight and temperature in the medium- and high-dose groups further underscores the ability of the microneedles to mitigate the systemic consequences of poisoning, such as metabolic stress and thermoregulatory dysfunction. In the first-aid setting, application of OPHDS5 microneedles 5 min after topical exposure significantly improved locomotor activity compared to untreated or blank microneedle controls, indicating that the enzyme can still access and neutralize the poison even after it has penetrated the skin. This suggests a window of opportunity for post-exposure intervention, which could be critical in scenarios where immediate prophylaxis is not feasible.

From a mechanistic standpoint, the efficacy of OPHDS5 delivered via microneedles can be attributed to its enzymatic hydrolysis of ethyl paraoxon at the dermal site. The rapid dissolution ensures that enzyme is available within minutes, coinciding with the time course of poison absorption through the skin. The local retention prevents systemic distribution, thereby avoiding dilution and potential degradation in the circulation.

Safety evaluation over 7 days post-application revealed no significant alterations in hematological or serum biochemical parameters, nor any histopathological abnormalities in major organs or skin. The slight fluctuation in ALT observed in the treated group was within the normal range and not accompanied by changes in other liver function markers, suggesting it is not clinically meaningful. These findings confirm the excellent biocompatibility of SCD-based microneedles and the absence of overt toxicity from OPHDS5 or the delivery system. The use of SCD, which is already approved in pharmaceutical formulations, further supports the translational potential of this approach.

Microneedles prepared from PVA, PVP, and SCD all exhibit good mechanical strength and have been widely used in microneedle fabrication. However, SCD microneedles exhibit better mechanical strength than PVA/PVP microneedles [25]. PVA solutions typically require heating to 80–90 °C for dissolution, and high-molecular-weight PVP also needs assisted heating; both materials have relatively long dissolution times. In contrast, SCD dissolves rapidly at room temperature, enabling fast drug release, which not only saves dissolution time but also prevents the inactivation of enzyme-based drugs caused by high temperatures.

Although the results are encouraging, several limitations of this study should be considered. First, this study was conducted only in rodent models. Regarding the patch size and dose for human use, we will perform pharmacokinetic extrapolations based on the effective dose in rats (a 15 × 15 array patch containing 1.2 mg of enzyme, covering a skin area of approximately 1 cm^2^) and human body surface area. Rodent data cannot be directly translated to humans, as differences in skin thickness, enzyme activity, and metabolic pathways between species may affect drug efficacy and safety profiles. Therefore, further pharmacodynamic studies and dose-ranging investigations in large animal models (e.g., swine) are necessary, and the translational value of this approach to humans remains to be validated. Second, in agricultural scenarios, dermal exposure to organophosphorus compounds is often localized (e.g., pesticide spray splashes, contaminated clothing). Applying a targeted patch directly to the exposed site is a convenient and feasible first-aid measure. For systemic poisoning caused by inhalation, microneedle delivery may need to be combined with systemic administration strategies such as intravenous or intramuscular injection. Third, this study focused only on ethyl paraoxon; the efficacy against other organophosphorus compounds requires further investigation. Fourth, the long-term stability of OPHDS5 within the microneedle matrix under storage conditions has not been evaluated, which is essential for practical application. Finally, the current manufacturing process is only at the laboratory scale; issues such as sterilization, packaging, and quality control need to be addressed for large-scale production before clinical translation. Future research should explore incorporating other OPH mutants with higher catalytic efficiency or broader substrate specificity, transitioning to large animal models such as swine, and combining the microneedle system with cholinesterase reactivators (oximes) to achieve synergistic effects, thereby further enhancing the applicability of this patch in human settings. The microneedle platform could also be adapted for co-delivery of multiple antidotes or for triggered release in response to specific stimuli (e.g., pH, enzymes). Furthermore, investigating the potential for immunogenicity of OPHDS5 upon repeated administration would be important for chronic use scenarios.

In summary, we have developed a fast-dissolving microneedle patch that effectively delivers OPHDS5 to the skin, providing rapid and sustained local protection against ethyl paraoxon poisoning. The system combines the advantages of rapid release, localized targeting, and excellent safety, offering a promising platform for transdermal delivery of protein-based therapeutics and for counteracting organophosphorus poisoning in both military and civilian settings. This approach has the potential to revolutionize the management of percutaneous poisoning by enabling self-administration and early intervention, thereby reducing morbidity and mortality.

## Data Availability

The original contributions presented in the study are included in the article, and further inquiries can be directed to the corresponding author.

## Supplementary Materials

The following supporting information can be downloaded at: https://www.mdpi.com/article/10.3390/pharmaceutics18050567/s1, Figure S1: Demolding images and optical microscope representative images of microneedles prepared with different materials (n = 4); Figure S2: Force-microneedle displacement curves for microneedles prepared with different concentrations of SCD; Figure S3: Parafilm after microneedle puncture; Figure S4: Body weight changes in SD rats among the three groups over 6 days post-challenge (n = 6); Figure S5: Body temperature changes in SD rats among the three groups over 6 days post-challenge (n = 6); Table S1: Properties of dissolvable microneedles prepared from different materials (n = 3).

## References

[1] Aroniadou-Anderjaska V., Apland J.P., Figueiredo T.H., Furtado M.D.A., Braga M.F. (2020). Acetylcholinesterase inhibitors (nerve agents) as weapons of mass destruction: History, mechanisms of action, and medical countermeasures. Neuropharmacology.

[2] Xu W., Zhao S., Zhang W., Wu H., Guang C., Mu W. (2021). Recent advances and future prospective of organophosphorus-degrading enzymes: Identification, modification, and application. Crit. Rev. Biotechnol..

[3] Rosenberg Y., Saxena A. (2020). Acetylcholinesterase inhibition resulting from exposure to inhaled OP can be prevented by pretreatment with BChE in both macaques and minipigs. Neuropharmacology.

[4] Ma M., Zhai Y., Di J., Gao X., Zheng A., Gao J. (2023). Research progress on organophosphorus compound scavengers and their delivery technologies. Chin. J. Pharmacol. Toxicol..

[5] Aztiria E., Baier C.J. (2025). Organophosphorus compounds and neurological conditions: Dr. Jekyll and Mr. Hyde. Neurotoxicol. Teratol..

[6] Xu Y., Chen X.F., He H.Q. (2026). Analysis of therapeutic effect of hemoperfusion on acute organophosphorus poisoning. Chin. J. Disaster Rescue Med..

[7] Wang Y., Jin X., Jiang X., Li F., Lin H., Yang X. (1984). Studies on protection against percutaneous absorption of toxic substances. Occup. Med..

[8] Wales M.E., Reeves T.E. (2012). Organophosphorus hydrolase as an in vivo catalytic nerve agent bioscavenger. Drug Test. Anal..

[9] Xi H., Liu C., Wen X., Zhao S. (2015). Research progress on the spatial structure and catalytic hydrolysis mechanism of phosphotriesterase. Chin. J. Appl. Environ. Biol..

[10] Tu C., Lei M., Ye L. (2021). Regulation of organophosphorus hydrolase nanocapsules and its effect on enzyme activity and stability. Chin. J. Biochem. Mol. Biol..

[11] Ma M., Zhai Y., Wang S., Zhang Z., Li Q., Gao J. (2024). Heterologous expression, purification, and preliminary evaluation of organophosphorus hydrolase against ethyl paraoxon poisoning. Chin. J. Pharmacol. Toxicol..

[12] Jaiswal S., Singh B., Dhingra I., Joshi A., Kodgire P. (2024). Bioremediation and bioscavenging for elimination of organophosphorus threats: An approach using enzymatic advancements. Environ. Res..

[13] Job L., Köhler A., Escher B., Worek F., Skerra A. (2020). A catalytic bioscavenger with improved stability and reduced susceptibility to oxidation for treatment of acute poisoning with neurotoxic organophosphorus compounds. Toxicol. Lett..

[14] Köhler A., Escher B., Job L., Koller M., Thiermann H., Skerra A., Worek F. (2021). Catalytic activity and stereoselectivity of engineered phosphotriesterases towards structurally different nerve agents in vitro. Arch. Toxicol..

[15] Goldsmith M., Eckstein S., Ashani Y., Greisen P., Leader H., Sussman J.L., Aggarwal N., Ovchinnikov S., Tawfik D.S., Baker D. (2016). Catalytic efficiencies of directly evolved phosphotriesterase variants with structurally different organophosphorus compounds in vitro. Arch. Toxicol..

[16] Tsai P.C., Fox N., Bigley A., Harvey S.P., Barondeau D.P., Raushel F.M. (2012). Enzymes for the homeland defense: Optimizing phosphotriesterase for the hydrolysis of organophosphate nerve agents. Biochemistry.

[17] Cherny I., Greisen P.J., Ashani Y., Khare S.D., Oberdorfer G., Leader H., Baker D., Tawfik D.S. (2013). Engineering V-Type Nerve Agents Detoxifying Enzymes Using Computa-tionally Focused Libraries. ACS Chem. Biol..

[18] Fang L., Wang L., Yao Y., Zhang J., Wu X., Li X., Wang H., Zhang X., Gong X., Chang J. (2017). Micro- and nano-carrier systems: The non-invasive and painless local administration strategies for disease therapy in mucosal tissues. Nanomedicine.

[19] Ito Y., Hagiwara E., Saeki A. (2007). Sustained-release self-dissolving micropiles for percutaneous absorption of insulin in mice. J. Drug Target..

[20] Jiskoot W., Randolph T.W., Volkin D.B., Middaugh C.R., Schöneich C., Winter G., Friess W., Crommelin D.J., Carpenter J.F. (2012). Protein instability and immunogenicity: Roadblocks to clinical application of injectable protein delivery systems for sustained release. J. Pharm. Sci..

[21] Sullivan S.P., Koutsonanos D.G., Del Pilar Martin M., Lee J.W., Zarnitsyn V., Choi S.-O., Murthy N., Compans R.W., Skountzou I., Prausnitz M.R. (2010). Dissolving polymer microneedle patches for influenza vaccination. Nat. Med..

[22] Chen Z., Li H., Bian Y., Wang Z., Chen G., Zhang X., Miao Y., Wen D., Wang J., Wan G. (2021). Bioorthogonal catalytic patch. Nat. Nanotechnol..

[23] Bae W.G., Ko H., So J.Y., Yi H., Lee C.-H., Lee D.-H., Ahn Y., Lee S.-H., Lee K., Jun J. (2019). Snake fang-inspired stamping patch for transdermal delivery of liquid formulations. Sci. Transl. Med..

[24] Yanagisawa Y., Nan Y., Okuro K., Aida T. (2018). Mechanically robust, readily repairable polymers via tailored noncovalent cross-linking. Science.

[25] Wang H., Fu Y., Mao J., Jiang H., Du S., Liu P., Tao J., Zhang L., Zhu J. (2022). Strong and tough supramolecular microneedle patches with ultrafast dissolution and rapid-onset capabilities. Adv. Mater..

[26] Lyu W.C. (2024). Spatiotemporal Orientation of Supramolecular Nanomicroneedles Induces Endogenous Ferroptosis in Keloids. Master’s Thesis.

[27] Wang H., Fu Y., Du S., Liu P., Ren J., Liu Y., Tao J., Zhang L., Zhu J. (2023). Mechanically robust dissolving microneedles made of supramolecular photosensitizers for effective photodynamic bacterial biofilm elimination. ACS Appl. Mater. Interfaces.

[28] Messner M., Kurkov S.V., Jansook P., Loftsson T. (2010). Self-assembled cyclodextrin aggregates and nanoparticles. Int. J. Pharm..

[29] Huang Z., Chen X., O’Neill S.J.K., Wu G., Whitaker D.J., Li J., McCune J.A., Scherman O.A. (2022). Highly compressible glass-like supramolecular polymer networks. Nat. Mater..

[30] Xu Y., Bian Q., Zhang Y., Zhang Y., Li D., Ma X., Wang R., Hu W., Hu J., Ye Y. (2025). Single-dose of integrated bilayer microneedles for enhanced hypertrophic scar therapy with rapid anti-inflammatory and sustained inhibition of myofibroblasts. Biomaterials.

[31] Zhang D., Gu Y., Zhang X., Cao W., Hou X., Ma J., Bao G. (2025). Study on detection methods for moisture content in liquid milk. China Dairy.

[32] Li Z. (2016). Preparation of Hyaluronic Acid Dissolving Microneedles and Their Application in Transdermal Tumor Therapy.

[33] Yin H., Tang W., Huang D., Gan J., Zhang P., Zhao Y., Bi Y. (2025). Bioinspired prolactin pulse release from responsive microneedles for inhibiting fatty liver formation. Adv. Sci..

[34] Wang Z., Li B., Nie C., Zhang R., Qu S., Shao Q., Zhang X., Li J., Li W., Li H. (2025). Photothermal conjugated polymer microneedle with biofilm elimination and angiogenesis for diabetic wound healing. Nano Lett..

[35] Yin Z. (2021). Preparation of rbFGF/CPC-Loaded Dissolving Microneedles and Their Therapeutic Effect on RAU.

[36] Liang S., Wu H., Wu X., Yuan W., Huang X. (2019). Preparation and performance study of fast-dissolving polymer microneedle patches. J. Chin. Med. Mater..

[37] Xue Q., Zhuang J., Liu F., Zhang X., Li M., Huang Y., Sun J., Xu H., Xu J. (2024). Preparation and performance evaluation of fast-dissolving polymer microneedles loaded with sitagliptin. Chin. J. Biotechnol..

[38] Yang J., Gong X., Zheng Y., Duan H., Chen S., Wu T., Yi C., Jiang L., Haick H. (2025). Microneedle-based integrated pharmacokinetic and pharmacodynamic evaluation platform for personalized medicine. Nat. Commun..

[39] Zhu L. (2021). Study on Dissolving Microneedles Loaded with Hydrophobic Drugs.

[40] Chen S., Wang J., Sun L., Xia F., Li W., Yuan L., Liu C., Li P., Bao C., Wang M. (2024). A quick paster type of soluble nanoparticle microneedle patch for the treatment of obesity. Biomaterials.

[41] Ma M., Zhai Y., Jin Q., Wang D., Wang S., Liu Z., Wang Z., Wang C., Xie Y., Ren Z. (2025). N-terminal PEGylation enhances organophosphorus hydrolase catalysis for a promising fast and long-acting prophylactic candidate. J. Hazard. Mater..

[42] Wan K., Song X., Shen L., Yang X., Shi J., Yang S., Qian S., Chen L. (2023). Research progress on construction methods of dissolving microneedles. J. Guizhou Univ. Tradit. Chin. Med..

[43] Schneider J.P., Ochs M. (2014). Alterations of mouse lung tissue dimensions during processing for morphometry: A comparison of methods. Am. J. Physiol.-Lung Cell. Mol. Physiol..

