# Supplementary material for: Fabrication and Evaluation of Rapidly Dissolving Microneedles Loaded with Organophosphorus Hydrolase for the Treatment of Transdermal Ethyl Paraoxon Poisoning
Source: Pharmaceutics. 2026 May 1;18(5):567. doi: 10.3390/pharmaceutics18050567 (PMC13210289; doi:10.3390/pharmaceutics18050567)
Supplement: Supplementary file 1 [file pharmaceutics-18-00567-s001.zip › pharmaceutics-4226132-supplementary.pdf]

Rapidly Dissolving Microneedles Loaded with Organophosphorus Hydrolase for Treatment of Organophosphate Poisoning

Fengqian Cui[1,2,3], Xue Liang[2,3]， Ming Ma[2,3], Yanan Zhai[2,3]*， Jing Gao[2,3]*

Affiliations

1. School of Pharmacy, Qingdao University, Qingdao, Shandong 266071, China
2. State Key Laboratory of National Security Specially Needed Medicines, Beijing 100039, China
3. Academy of Military Medical Sciences, Beijing 100850, China

***Corresponding authors.**

Email addresses: zyn1989@mail.ustc.edu.cn (Y. Zhai); gjsmmu@126.com (J. Gao).

Supplementary information

Table of Contents

Supplementary Figures S1 to S5

Supplementary Table S1

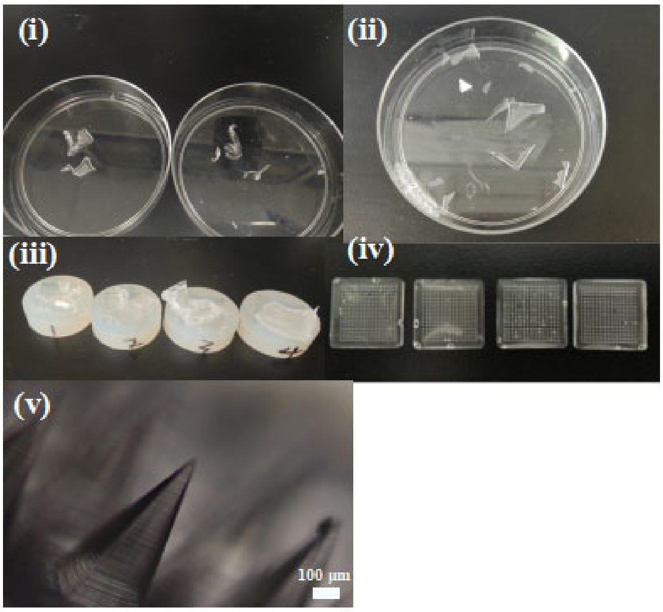

Supplementary Figure S1 Demolding images and optical microscope representative images of microneedles prepared with different materials (n = 4). (i) Demolding image of trehalose microneedles; (ii) Demolding image of dextran 20 microneedles; (iii) Demolding image of maltose microneedles; (iv) Demolding image of SCD microneedles; (v) Optical microscope representative image of SCD microneedles (scale bar: 100 μm).

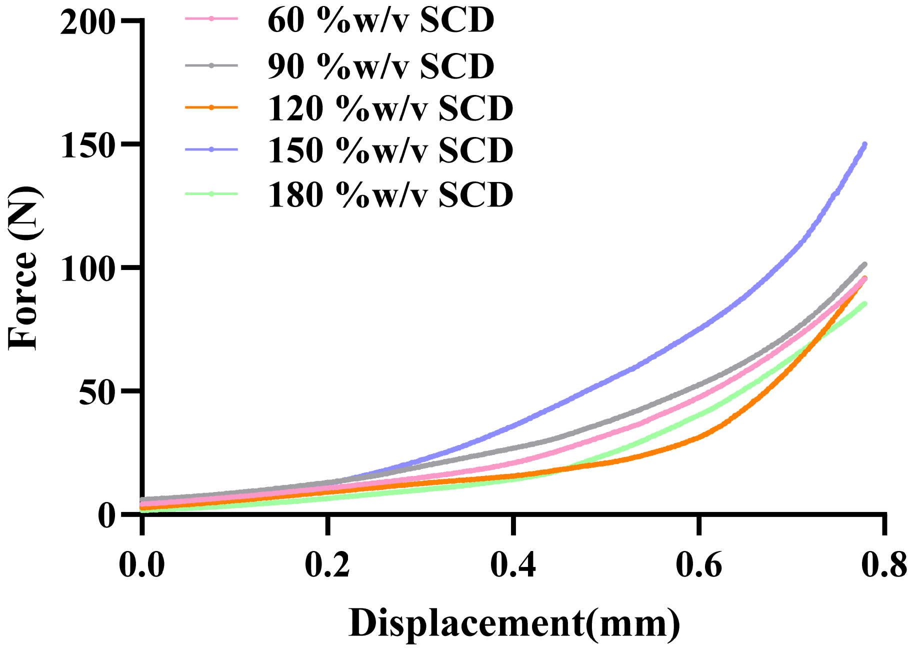

Supplementary Figure S2 Force-microneedle displacement curves for micro ne-edles prepared with different concentrations of SCD.

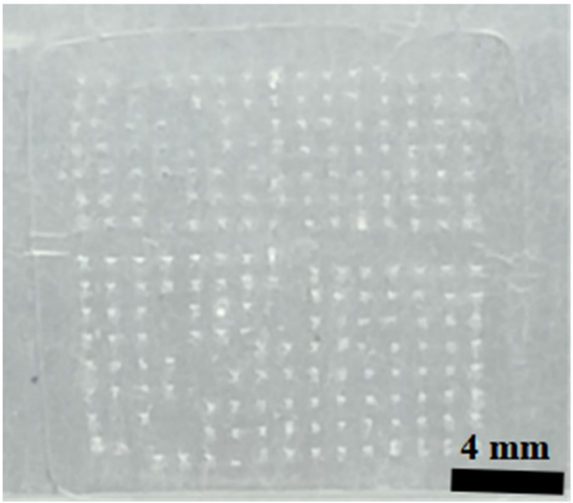

Supplementary Figure S3 Parafilm after microneedle puncture.

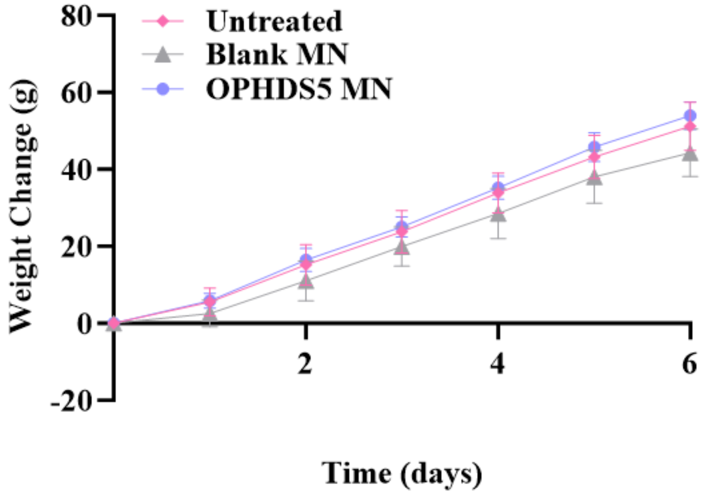

Supplementary Figure S4 Body weight changes in SD rats among the three groups over 6 days post-challenge (n = 6).

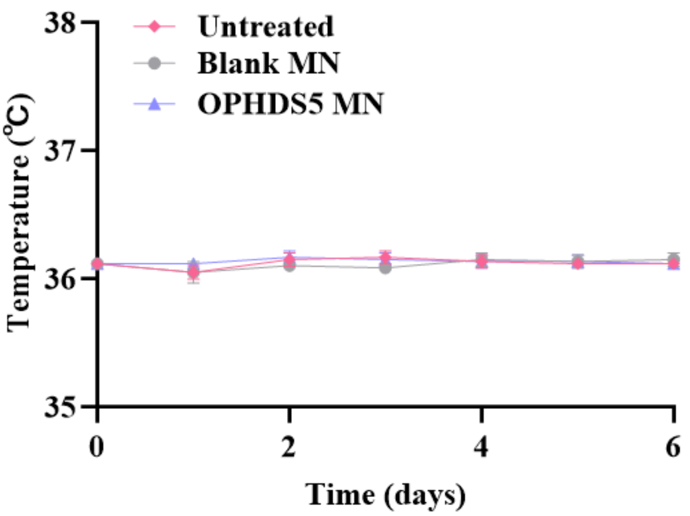

Supplementary Figure S5 Body temperature changes in SD rats among the three groups over 6 days post-challenge (n = 6).

Supplementary Table S1. Properties of dissolvable microneedles prepared fr o-m different materials（n = 3）.

Microneedle material	Solution viscosity（mPa·S）	Forming status
6 %w/v trehalose	1.54 ± 0.01	Flaky, difficult to demould
8 %w/v trehalose	2.12 ± 0.01	Flaky, difficult to demould
10 %w/v trehalose	2.42 ± 0.01	Flaky, difficult to demould
10 %w/v Dextran-20	0.89 ± 0.03	Flaky shape, relatively difficult to demould
15 %w/v Dextran-20	2.08 ± 0.02	Flaky shape, relatively difficult to demould
20 %w/v Dextran-20	2.97 ± 0.08	Flaky shape, relatively difficult to demould
20 %w/v maltose	2.28 ± 10.02	Flaky shape, relatively difficult to demould
40 %w/v maltose	3.96 ± 3.06	Flaky shape, relatively difficult to demould
60 %w/v maltose	6.22 ± 19.76	Flaky shape, relatively difficult to demould
80 %w/v maltose	11.62 ± 7.55	Flaky shape, relatively difficult to demould
100 %w/v maltose	22.26 ± 29.69	Flaky shape, relatively difficult to demould
60 %w/v SCD	7.32 ± 0.02	Clear needle tips, easy demolding
90 %w/v SCD	18.20 ± 0.01	Clear needle tips, easy demolding
120 %w/v SCD	62.63 ± 0.23	Clear needle tips, easy demolding
150 %w/v SCD	226.93 ± 0.60	Clear needle tips, easy demolding
180 %w/v SCD	696.53 ± 5.70	Clear needle tips, easy demolding
